# Diet Quality Scores and Asthenoteratozoospermia Risk: Finding From a Hospital-Based Case–Control Study in China

**DOI:** 10.3389/fnut.2022.859143

**Published:** 2022-04-11

**Authors:** Qi Cui, Hui-Han Wang, Qi-Jun Wu, Xiao-Bin Wang, Ren-Hao Guo, Xu Leng, Xiao-Ling Tan, Qiang Du, Bo-Chen Pan

**Affiliations:** ^1^Department of Frigidzone Medicine, College of High Altitude Military Medicine, Army Medical University (Third Military Medical University), Chongqing, China; ^2^Key Laboratory of Extreme Environmental Medicine, Ministry of Education of China, Chongqing, China; ^3^Key Laboratory of High Altitude Medicine, People's Liberation Army (PLA), Chongqing, China; ^4^Department of Hematology, Shengjing Hospital of China Medical University, Shenyang, China; ^5^Department of Clinical Epidemiology, Shengjing Hospital of China Medical University, Shenyang, China; ^6^Clinical Research Center, Shengjing Hospital of China Medical University, Shenyang, China; ^7^Center of Reproductive Medicine, Shengjing Hospital of China Medical University, Shenyang, China

**Keywords:** DASH, AHEI-2010, CHEI, asthenoteratozoospermia, case–control study

## Abstract

**Objective:**

We aimed to examine associations of diet quality scores, including the dietary approaches to stop hypertension (DASH), alternate Healthy Eating Index (AHEI), and Chinese Healthy Eating Index (CHEI) with asthenoteratozoospermia risk in China.

**Methods:**

Among 254 cases and 633 controls in a hospital-based case–control study in Shenyang, Liaoning Province, China, DASH, AHEI, and CHEI were calculated using a validated food frequency questionnaire. Asthenotetrazoospermia was evaluated according to World Health Organization guidelines. Unconditional multiple logistic regression was used to estimate odds ratios (ORs) with 95% confidence intervals (CIs) for the association between quality diet scores and asthenoteratozoospermia risk.

**Results:**

We found that the CHEI score was inversely associated with asthenoteratozoospermia risk, with ORs of 0.59 (95% CI 0.39, 0.88) and 0.59 (95% CI 0.39, 0.88) for the 2nd and 3rd tertiles vs. the 1st tertile, respectively (*P* trend < 0.05). In addition, our data indicated that each standard deviation increase in CHEI, AHEI-2010, and DASH score was associated with 19, 13, and 17% decreased risk of asthenoteratozoospermia, respectively.

**Conclusion:**

Our findings suggest that higher adherence to the CHEI, AHEI-2010, and DASH diet quality scores may reduce the risk of asthenoteratozoospermia, especially for younger participants.

## Introduction

According to the WHO estimation, fertility problems have become an increasing medical problem affecting ~60–80 million couples, and male factors account for almost 50% of cases (1). As one of the principal types, asthenoteratozoospermia is defined as low sperm vitality or even malformation (2). In addition to genetic factors, environmental factors, lifestyle habits, and dietary factors may be relevant to sperm quality (3). Epidemiological studies have reported that sweetened beverages, red meat, and organ meat consumption are negative and that fish, egg, nut, and low-fat dairy consumption are positively correlated with sperm quality (4–7).

In reality, however, individuals eat combinations of food as meals instead of a single food, which makes it difficult to distinguish their individual effects (8). To overcome this challenge, dietary patterns were used to assess the effect of overall diet on sperm quality. A meta-analysis reported that individuals with the highest adherence to the healthy dietary pattern (highly related to fruit, vegetables, tomatoes, whole grain, legumes, and fish) had a significantly higher level of sperm concentration than those who had the lowest adherence to the healthy dietary pattern (9). Given that these dietary patterns (data-driven) do not apply to another population, the diet quality scores (priori-defined) are easier to calculate and compare across studies (10).

Dietary inflammatory index (DII) scores, as one of a priori-defined score, have been reported to not be associated with asthenozoospermia risk in our previous study (11). Other priori-defined diet scores have been reported to associate with overall sperm quality, such as the Healthy Eating Index-2005 (HEI-2005), the Alternate Healthy Eating Index-2010 (AHEI-2010), and dietary approaches to stop hypertension (DASH) (1, 12, 13). Studies have shown that HEI, AHEI, and DASH diet scores were associated with better overall sperm quality in Israel (1), and adherence to the DASH diet scores was related to a higher sperm count and concentration in southern Spain and Poland (12, 13). However, it is unknown whether this association can be extrapolated to Chinese populations. In addition, the relationships between the Chinese Healthy Eating Index (CHEI), which is used to assess overall diet quality among Chinese populations (14), and overall sperm quality have been unclear.

Therefore, to explore the diet quality scores appropriated for the Chinese population that can be used as a tool to improve sperm quality, we conducted a hospital–based case–control study to investigate the associations between DASH, AHEI-2010, and CHEI diet quality scores and asthenoteratozoospermia risk in China.

## Materials and Methods

### Design and Population

**Figure 1:**
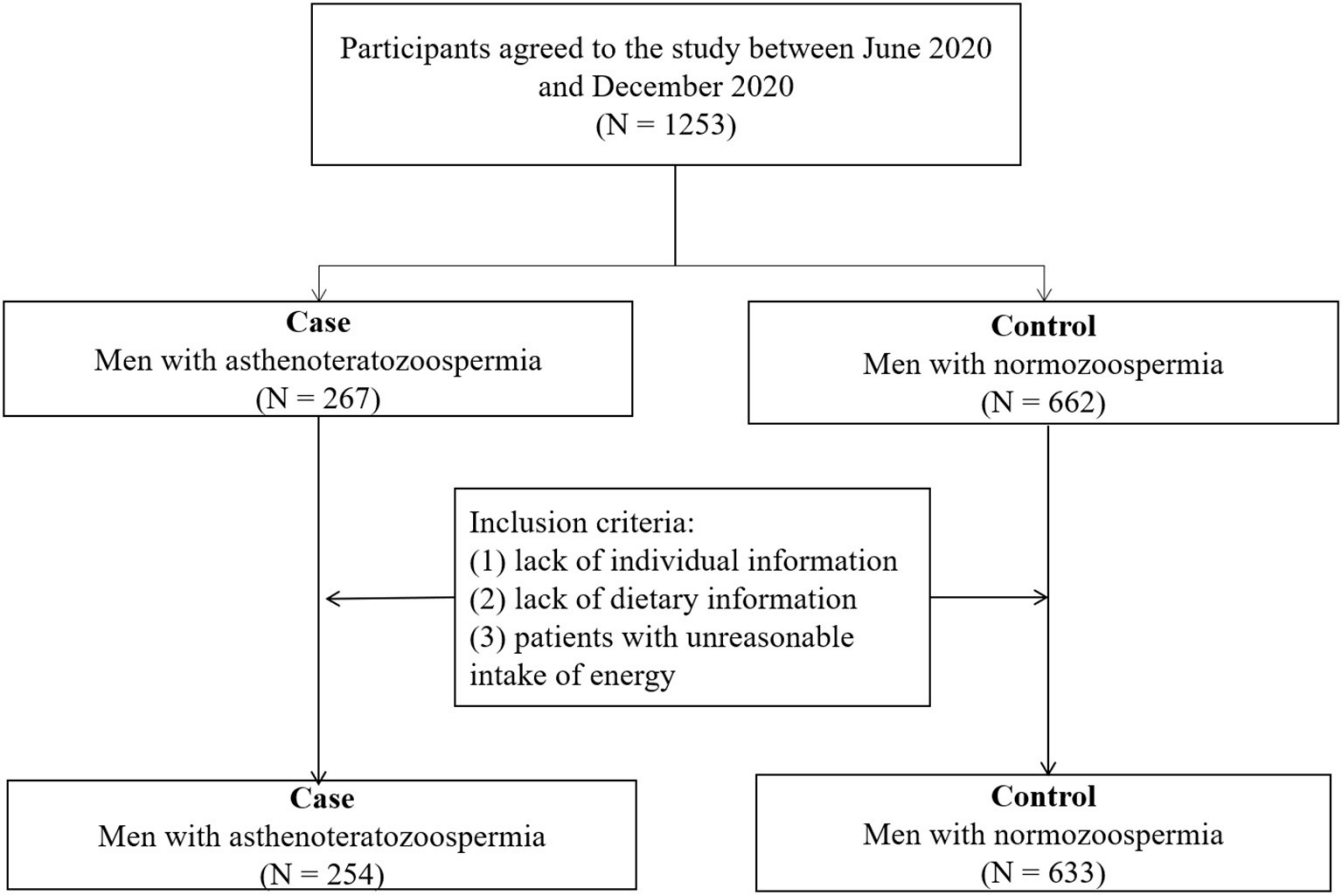
Flow chart of participants.

The present hospital-based case–control study was conducted to compare the diet quality scores of men with and without asthenoteratozoospermia. Study participants were men referred to the infertility clinic of Shengjing Hospital of China Medical University from June 2020 to December 2020. As shown in Figure 1, a reproductive medicine specialist diagnosed each man with asthenoteratozoospermia as a new patient (incident case) and was placed in the case group (*n* = 267) based on the World Health Organization (WHO) laboratory manual for the examination and processing of human semen (fifth edition, 2010) (1). Asthenoteratozoospermia is defined as the concentration and proportions of motile and morphologically normal spermatozoa below the reference values (<15 × 10^6^ sperm/mL, <32% progressive motility, and <4% normal morphology) (1). Men with normozoospermia (*n* = 662) were selected as the control group and were companions of patients admitted to the same infertility clinic. Trained research interviewers administered a baseline questionnaire that included sociodemographic and dietary history for recruited men. Patients who had missing individual information or an invalid food frequency questionnaire (FFQ) were not eligible for recruitment, nor were patients with unreasonable intake of energy (<800 or >4,200 kcal/d). Two hundred fifty-four cases with asthenoteratozoospermia and 633 controls were included in the final analysis.

Ethical and research approvals were obtained from the ethics committee of Shengjing Hospital of China Medical University, with all participants providing written consent at study enrollment.

### Semen Collection and Analysis

For 3–7 days of celibate, semen samples were collected without using condoms or lubricants through masturbation into a plastic tube in a dedicated room. Samples were liquefied for 60 min before analysis. Ejaculate volume, pH, sperm concentration, total count of sperm inspected, total motility, and the percentage of each motile grade of sperm were measured with WLJY9000. Flow cytometry was used to assess sperm DNA fragmentation and sperm DNA staining. After pasteurization, sperm smears were observed under an optical microscope and evaluated for morphology. The reference values of normal sperm were determined by the WHO criteria (15). External quality was controlled by experienced technicians.

### Data Collection

All participants were asked to complete a self-administered, semiquantitative validated food frequency questionnaire (FFQ) that included 110 food items: they were asked how often, on average, during the previous year they had consumed each food. Estimations of energy and nutrient intakes were performed with the aid of the Chinese Food Composition Tables (16). In addition, demographic characteristics (age, education level, and household income), lifestyle (smoking, drinking, and physical activity), and other information were collected by a questionnaire. The heights and weights were collected by physical examination. After obtaining these measurements, the body mass index (BMI) was taken for each participant by dividing weight (kg) by height squared (m^2^).

### Diet Quality Scores

We assessed 3 diet quality scores, and details are presented in Supplementary Table 1. The DASH score includes eight components: fruits, vegetables, nuts/legumes, dairy, whole grains, red meat, sugar-sweetened beverages, and sodium intake, which range from 8 to 40 (17). The AHEI-2010 score is based on vegetables (excluding potatoes), fruits, whole grains, nuts and legumes, long-chain n-3 fatty acids, polyunsaturated fatty acids, sweetened beverages and juice, red and processed meat, trans fat, sodium, and alcoholic drinks, which range from 1 to 110 (18). The CHEI score is calculated according to 12 adequacy components (total grains, whole grains and mixed beans, tubers, total vegetables, dark vegetables, fruits, dairy, soybeans, fish and seafood, seeds and nuts, poultry, and eggs) and 5 moderation components (red meat, cooking oils, sodium, added sugars, and alcohol), which range from 0 to 100 (14).

### Statistical Analysis

Descriptive statistics were examined for all variables. Continuous variables are reported as the mean ± standard deviation; categorical variables are presented as numbers of participants and percentages. We conducted Student's *t*-tests or chi-square-tests to compare differences between groups. We created tertiles of each diet score and used unconditional logistic regression analyses to estimate odds ratios (ORs) with 95% confidence intervals (CIs) to assess associations between quality scores and asthenoteratozoospermia risk. The lowest tertile of scores was considered the reference group. A linear trend was tested by treating the median value of each tertile as a continuous variable. In addition, we alternatively assessed the risk estimates associated with a continuous measure for a 1 standard deviation (SD) increase in scores. We used three models: model 1 (adjusted for energy and age) and model 2 (adjusted for energy, age, smoking status, drinking status, household income, education level, abstinence time, and physical activity). We conducted adjusted risk estimates for asthenoteratozoospermia by CHEI, AHEI-2010 and DASH scores in subgroup analyses stratified by age, BMI, and smoking status.

All statistical analyses were performed using SAS software, version 9.3, for Windows (SAS Institute Inc., Cary, NC, USA). All reported *P*-values are two-sided, and those that were <0.05 were defined as statistically significant.

## Results

### Characteristics of the Participants

As shown in Table 1, cases were more likely to have longer abstinence times and a higher proportion of current smokers than controls. In addition, the case with asthenoteratozoospermia tended to have a lower sperm concentration, total sperm count, progressive motility, total motility and normal sperm morphology than controls.

**Table 1:** General characteristics of participants in a case-control study of asthenoteratozoospermia.

**Characteristics**	**Normal**	**Asthenoteratozoospermia**	P^*^
**No. of participants**	633	254	
**Age (years)**	32.11 ± 4.52	34.05 ± 5.67	<0.01
**Body mass index (kg/m** ^**2**^ **)**	26.17 ± 4.51	26.03 ± 3.96	0.64
**Physical activity (MET/hours/week)**	165.46 ± 101.34	166.17 ± 111.78	0.93
**Abstinence time (days)**	4.29 ± 1.39	4.49 ± 1.48	<0.05
**Energy intake (kcal/day)**	1,767.03 ± 562.71	1,809.37 ± 576.51	0.31
**Educational level (** ***n*** **, %)**			0.11
Middle school or below	243 (38.39)	83 (32.68)	
College or higher	390 (61.61)	171 (67.32)	
**Annual family income (RMB, thousand yuan) (** ***n*** **, %)**			0.69
<50	99 (15.64)	34 (13.39)	
50 to <100	242 (38.42)	100 (39.37)	
≥100	292 (46.13)	120 (47.24)	
**Current smoker (** ***n*** **, %)**	334 (52.76)	109 (42.91)	<0.01
**Current drinker (** ***n*** **, %)**	272 (42.97)	93 (36.61)	0.09
**Semen parameters**			
Ejaculate volume (ml)	3.45 ± 1.26	3.72 ± 1.64	<0.05
Sperm concentration (10^6^/ml)	70.52 ± 39.79	47.38 ± 36.45	<0.01
Total sperm count (10^6^/ml)	230.78 ± 133.01	172.81 ± 161.86	<0.01
Progress motility (%)	44.39 ± 9.34	17.95 ± 8.86	<0.01
Total motility (%)	54.75 ± 11.35	22.71 ± 10.89	<0.01
Normal sperm morphology (%)	6.67 ± 2.72	1.82 ± 1.25	<0.01

*MET, metabolic equivalent*.

*Values are presented as mean ± SD (continuous variables) or as n and % (categorical variables)*.

* *P-value was determined by Student's t-test or chi-square-test*.

General characteristics of participants according to tertiles of CHEI, DASH, and AHEI-2010 scores are presented in Supplementary Tables 2–4. Semen parameters, progress motility, and total motility were all increased across tertiles for CHEI, AHEI-2010, and DASH scores (*P* < 0.05). Participants with a high score of these three diet patterns were more likely to be younger, consume less energy and have a smoking and drinking history (*P* < 0.05).

**Table 2:** Adjusted OR and 95 % CIs for asthenoteratozoospermia risk according to tertile of dietary quality scores.

	**Tertiles of scores**			***P*** **for trend**	**Per SD increment**
	**T1**	**T2**	**T3**		
**CHEI score**					
Range of scores	<60.99	60.99 to <69.41	≥69.41		
No. of case/control	109/211	94/211	51/211		
Model 1^a^	1.00 (Ref)	0.55 (0.37, 0.81)	0.53 (0.36, 0.79)	<0.01	0.78 (0.67, 0.91)
Model 2^b^	1.00 (Ref)	0.59 (0.39, 0.88)	0.59 (0.39, 0.88)	<0.05	0.81 (0.69, 0.96)
**AHEI-2010 score**					
Range of scores	<29.65	29.65 to <36.79	≥36.79		
No. of case/control	112/211	82/211	60/211		
Model 1^a^	1.00 (Ref)	0.81 (0.55, 1.19)	0.63 (0.43, 0.93)	0.06	0.83 (0.71, 0.98)
Model 2^b^	1.00 (Ref)	0.86 (0.58, 1.28)	0.70 (0.47, 1.03)	0.17	0.87 (0.73, 1.03)
**DASH score**					
Range of scores	<22.00	22.00 to <26.00	≥26.00		
No. of case/control	119/211	59/211	76/211		
Model 1^a^	1.00 (Ref)	0.92 (0.67, 1.29)	0.72 (0.52, 1.00)	0.10	0.79 (0.67, 0.94)
Model 2^b^	1.00 (Ref)	0.97 (0.70, 1.36)	0.78 (0.56, 1.10)	0.29	0.83 (0.70, 0.99)

*AHEI, Alternate Healthy Eating Index; CHEI, Chinese Healthy Eating Index; DASH, Dietary Approach to Stop Hypertension; ORs (95% CI), Odds ratios (95% confidence interval); Ref, reference category; SD, standard deviation*.

a *Adjusted for total energy intake and age*.

b *Adjusted for total energy intake, age, body mass index, smoking status, drinking status, household income, education level, abstinence time and physical activity*.

### Association of Diet Quality With Asthenoteratozoospermia Risk

The relationships between diet quality scores and asthenoteratozoospermia risk are presented in Table 2. The CHEI score was inversely associated with the risk of asthenoteratozoospermia in the age- and total energy-adjusted model (T3 vs. T1, OR = 0.53, 95% CI: 0.36–0.79, *P* trend < 0.01) as well as in the multivariate adjusted model (T3 vs. T1, OR = 0.59, 95% CI: 0.39–0.88, *P* trend < 0.05). In addition, we found no statistically significant associations between AHEI-2010 and DASH and asthenoteratozoospermia risk in the age- and total energy-adjusted models (T3 vs. T1, OR = 0.63, 95% CI: 0.43–0.93, *P* trend = 0.06 for AHEI-2010; OR = 0.72, 95% CI: 0.52–1.00, *P* trend = 0.10 for DASH) or multivariable-adjusted models (T3 vs. T1, OR = 0.70, 95% CI: 0.47–1.03, *P* trend = 0.17; OR = 0.78, 95% CI: 0.56–1.10, *P* trend = 0.29). We found that these diet quality scores were significantly associated with the decreased risk of asthenoteratozoospermia by 22% (95% CI, 9–33%) for per 1 SD increase in CHEI score, 17% (95% CI, 2–29%) for per 1 SD increase in AHEI score, and 21% (95% CI, 6–33%) for per 1 SD increase in DASH score.

### Stratified Analyses

As shown in Table 3, we found that there were significant interactive effects of age and smoking status on the associations (all *P* for interaction < 0.05). For these scores, significant reverse associations were only witnessed in younger patients (T3 vs. T1, OR = 0.47, 95% CI: 0.28–0.77 for CHEI; OR = 0.51, 95% CI: 0.31–0.84, *P* trend < 0.05 for AHEI-2010; OR = 0.54, 95% CI: 0.33–0.88, *P* trend < 0.05 for DASH). When conducting subgroup analysis by smoking status, we found that CHEI was negatively related to asthenoteratozoospermia risk only in non-smokers (T3 vs. T1, OR = 0.43, 95% CI: 0.23–0.77).

**Table 3:** Adjusted OR and 95 % CIs for asthenoteratozoospermia risk by subgroup analyses^a^.

	**Tertiles of scores**			***P*** **for trend**	***P*** **for interaction**
	**T1**	**T2**	**T3**		
**CHEI score**					
**Age (years)**					
≤ 32	1.00 (Ref)	0.63 (0.37, 1.05)	0.47 (0.28, 0.77)	<0.05	<0.01
>32	1.00 (Ref)	0.56 (0.29, 1.03)	0.81 (0.40, 1.63)	0.31	
**BMI (kg/m** ^**2**^ **)**					
<25	1.00 (Ref)	0.64 (0.35, 1.17)	0.56 (0.30, 1.04)	0.31	0.90
≥25	1.00 (Ref)	0.54 (0.31, 0.91)	0.48 (0.28, 0.81)	<0.05	
**Current smoker**					
Yes	1.00 (Ref)	0.67 (0.39, 1.15)	0.59 (0.33, 1.05)	0.30	<0.05
No	1.00 (Ref)	0.49 (0.26, 0.89)	0.43 (0.23, 0.77)	<0.05	
**AHEI-2010 score**					
**Age (years)**					
≤ 32	1.00 (Ref)	0.83 (0.49, 1.38)	0.51 (0.31, 0.84)	<0.05	<0.01
>32	1.00 (Ref)	0.80 (0.43, 1.49)	1.05 (0.53, 2.08)	0.86	
**BMI (kg/m** ^**2**^ **)**					
<25	1.00 (Ref)	0.71 (0.39, 1.26)	0.82 (0.44, 1.52)	0.71	0.63
≥25	1.00 (Ref)	0.85 (0.50, 1.45)	0.48 (0.29, 0.80)	<0.05	
**Current smoker**					
Yes	1.00 (Ref)	0.79 (0.46, 1.38)	0.54 (0.31, 0.96)	0.21	<0.05
No	1.00 (Ref)	0.75 (0.42, 1.31)	0.59 (0.34, 1.01)	0.28	
**DASH score**					
**Age (years)**					
≤ 32	1.00 (Ref)	0.83 (0.49, 1.41)	0.54 (0.33, 0.88)	<0.05	<0.01
>32	1.00 (Ref)	0.88 (0.46, 1.74)	1.09 (0.57, 2.07)	0.95	
**BMI (kg/m** ^**2**^ **)**					
<25	1.00 (Ref)	0.89 (0.48, 1.67)	0.77 (0.42, 1.42)	0.70	0.53
≥25	1.00 (Ref)	0.80 (0.46, 1.39)	0.60 (0.36, 0.97)	0.11	
**Current smoker**					
Yes	1.00 (Ref)	0.99 (0.55, 1.82)	0.58 (0.33, 1.01)	0.22	<0.05
No	1.00 (Ref)	0.70 (0.39, 1.26)	0.68 (0.39, 1.14)	0.50	

*AHEI, Alternate Healthy Eating Index; CHEI, Chinese Healthy Eating Index; DASH, Dietary Approach to Stop Hypertension; ORs (95% CI), Odds ratios (95% confidence interval); Ref, reference category*.

^a^*Adjusted for total energy intake, age, body mass index, smoking status, drinking status, household income, education level, abstinence time and physical activity*.

## Discussion

In the current study, we found that the inverse association between diet quality scores and asthenoteratozoospermia risk was evident for CHEI, AHEI-2010 and DASH scores overall, when the SD of diet quality scores was considered a continuous variable. In the stratified analyses, significant inverse associations for these three diet scores only appeared for individuals at a younger age.

There has been no study investigating the association between multiple diet quality scores and asthenoteratozoospermia risk. We found that higher adherence to the AHEI-2010 and DASH scores was inversely associated with asthenoteratozoospermia risk. Our results are partially consistent with a cross-sectional study conducted in Poland that reported that adherence to AHEI-2010 and DASH scores may have higher sperm concentration, normal sperm morphology, and total sperm count (12). Some similarities may also be found with observations conducted in Israel reporting that men in the highest quartiles of AHEI-2010 and DASH scores had significantly higher sperm concentrations and normal sperm morphology (1).

The AHEI-2010 and DASH scores were characterized by high intakes of fruit, vegetables, whole grains, and legumes and low intakes of sugar-sweetened, red/processed meat, and sodium. These high intake food groups were found to be positively associated with sperm count (3, 19), concentration (19), sperm motility (20, 21), and morphology (3, 19). These food groups serve as major sources of vitamins, minerals, and polyphenols, which are be considered antioxidant and anti-inflammatory. The antioxidants in male reproductive health may be positively associated with semen parameters (22–24), as these oxidants may reduce oxidative damage to sperm (25, 26), DNA maintenance, transfer RNA, and protein synthesis (27), and reduce the negative effects of inflammation (26, 28). Moreover, these two dietary scores recommend reduction in sugar-sweetened beverage, red/processed meat, and fat consumption. The relatively high content of sugars, saturated/trans fatty acids, and sodium may adversely affect sperm count (19), concentration (29, 30), sperm motility (3, 20), and morphology (3, 31).

In addition, we found that CHEI scores were inversely associated with asthenoteratozoospermia risk. As the instrument in China to assess diet quality overall, the CHEI score was developed according to the updated Dietary Guidelines for Chinese (14). The main components recommended by the CHEI score are higher intakes of total grains, whole grains, and mixed beans, tubers, total vegetables, dark vegetables, fruits, dairy, soybeans, fish and seafood, poultry, eggs, seeds, and nuts and limitation of red meat, cooking oils, sodium, added sugars and alcohol (14). Evidence from epidemiological studies has reported that high intakes of dairy (4), fruits and vegetables (19, 32), cereals (32), fish (5, 6), nuts (6), and eggs (6) were related to higher sperm quality. In addition, high consumption of red meat (6, 32), processed meat (5), habitual alcohol consumption (33), sugar-sweetened beverages (34), and fish was found to be associated with low semen quality. The apparent benefit of CHEI may be attributable to a high intake of foods containing antioxidants and carotenoids. Reactive oxygen species production has been associated with increased cellular damage and the rate of sperm ATP depletion, which leads to insufficient axonemal phosphorylation, lipid peroxidation, and loss of motility and viability (35). In addition, the CHEI pattern also contains a high amount of nutrients with good anti-inflammatory properties, such as omega-3 fatty acids, fruit, and vegetables, and a low amount of proinflammatory nutrients, such as red meat and unhealthy fat. Inflammation may affect reproduction through anatomical or functional changes in the male accessory gland and/or direct negative impacts on spermatozoa (28).

In the stratified analyses, we found that the favorable associations between the three diet quality scores and asthenoteratozoospermia risk persisted across participants whose age was below 32 years, which indicated that adhering to the latest dietary guidelines for Americans or Chinese individuals is more likely to decrease asthenoteratozoospermia risk for young participants. The reason may be that young participants often show less healthy eating habits than general participants (36, 37). In addition, smoking can have a harmful effect on semen quality through a variety of toxic substances and chemical substances (38). However, the beneficial substances produced by diet cannot compensate for the harm to semen quality caused by smoking, which may explain the negative correlations between the CHEI score and asthenoteratozoospermia risk in non-smokers.

Our study has several strengths. To our knowledge, this is the first study to investigate the association between CHEI, AHEI-2010, and DASH and the risk of asthenoteratozoospermia in a Chinese population. Furthermore, we not only adjusted for several confounding factors but also carried out numerous subgroup analyses, which ensured the authenticity and reliability of our research results. However, some limitations occurred in our study. First, we cannot exclude the possibility of reverse causality with a case–control design. However, we provided strong evidence of a positive association by eliminating the effect of potential confounders. Second, dietary information collected by the FFQ may induce recall bias. To reduce this bias, we used the FFQ, which has acceptable reproducibility and validity, to collect dietary information in a face-to-face manner. Third, there are some dietary components that are not included in the diet score, such as oil and salt, which may affect our results. In the future, we need to consider these factors to improve these studies.

Our findings suggested that higher adherence to the CHEI, AHEI-2010, and DASH diet quality scores may reduce the risk of asthenoteratozoospermia, especially for younger participants. Well-designed prospective cohort studies and randomized clinical trials are required to confirm these results.

## Data Availability Statement

The original contributions presented in the study are included in the article/Supplementary Material, further inquiries can be directed to the corresponding authors.

## Ethics Statement

The studies involving human participants were reviewed and approved by the Ethics Committee of Shengjing Hospital of China Medical University. The patients/participants provided their written informed consent to participate in this study.

## Author Contributions

QC, H-HW, Q-JW, QD, and B-CP conceived the study. B-CP contributed to the design. QD, X-BW, R-HG, XL, and B-CP collected the data. QC and Q-JW cleaned the data, checked the discrepancy, and analyzed the data. QC, H-HW, Q-JW, X-BW, R-HG, XL, X-LT, and B-CP interpreted the data. All authors read the manuscript and approved the final vision.

## Funding

This work was supported by the Shengjing Hospital Clinical Research Project (No. M0071 to B-CP).

## Conflict of Interest

The authors declare that the research was conducted in the absence of any commercial or financial relationships that could be construed as a potential conflict of interest.

## Publisher's Note

All claims expressed in this article are solely those of the authors and do not necessarily represent those of their affiliated organizations, or those of the publisher, the editors and the reviewers. Any product that may be evaluated in this article, or claim that may be made by its manufacturer, is not guaranteed or endorsed by the publisher.
