# Supplementary material for: Diet Quality Scores and Asthenoteratozoospermia Risk: Finding From a Hospital-Based Case–Control Study in China
Source: Front Nutr. 2022 Apr 11;9:859143. doi: 10.3389/fnut.2022.859143 (PMC9036176; doi:10.3389/fnut.2022.859143)
Supplement: Supplementary file 1 [file Table_1.DOCX]

Supplementary Material

# Supplementary Table 1. Criteria for scoring CHEI, AHEI-2010, and DASH scores

| **Component** | **CHEI ^a^** | | | **AHEI-2010 ^b^** | | | **DASH ^c^** | | |
| --- | --- | --- | --- | --- | --- | --- | --- | --- | --- |
|  | **Point** | **Max** | **Min** | **Point** | **Max** | **Min** | **Point** | **Max** | **Min** |
| Total grains | 5 | ≥2.5SP/1000 kcal | 0 |  |  |  |  |  |  |
| Whole Grains/mixed beans | 5 | ≥0.6SP/1000 kcal | 0 |  |  |  |  |  |  |
| Whole Grains |  |  |  | 10 | 90 g/d | 0 | 5 | Q5 | Q1 |
| Tubers | 5 | ≥0.3SP/1000 kcal | 0 |  |  |  |  |  |  |
| Total vegetables | 5 | ≥1.9SP/1000 kcal | 0 | 10 | **≥**5 servings/d | 0 | 5 | Q5 | Q1 |
| Dark vegetables | 5 | ≥0.9SP/1000 kcal | 0 |  |  |  |  |  |  |
| Total fruits | 10 | ≥1.1SP/1000 kcal | 0 | 10 | **≥**4 servings/d | 0 | 5 | Q5 | Q1 |
| Dairy | 5 | ≥0.5SP/1000 kcal | 0 |  |  |  | 5 | Q5 | Q1 |
| Soybeans | 5 | ≥0.4SP/1000 kcal | 0 |  |  |  |  |  |  |
| Fish and Seafood | 5 | ≥0.4SP/1000 kcal | 0 |  |  |  |  |  |  |
| Poultry | 5 | ≥0.3SP/1000 kcal | 0 |  |  |  |  |  |  |
| Eggs | 5 | ≥0.5SP/1000 kcal | 0 |  |  |  |  |  |  |
| Seeds and Nuts | 5 | ≥0.4SP/1000 kcal | 0 | 10 | **≥**1 servings/d | 0 | 5 | Q5 | Q1 |
| Sugar-sweetened beverages ^d^ |  |  |  | 10 | 0 | **≥**1 servings/d | 5 | Q1 | Q5 |
| Added sugars | 5 | ≤10% of energy | ≥20% of energy |  |  |  |  |  |  |
| Sodium ^e^ | 10 | 1 point | 5 points | 10 | 1 point | 5 points | 5 | 1 point | 5 points |
| Cooking oils ^f^ | 10 | 1 point | 5 points |  |  |  |  |  |  |
| Red/processed meat |  |  |  | 10 | 0 | **≥**1.5 servings/d | 5 | Q1 | Q5 |
| Red meat | 5 | ≤0.4SP/1000 kcal | ≥3.5SP/1000 kcal |  |  |  |  |  |  |
| Alcohol | 5 | ≤15 g | ≥40g | 10 | 0.5–2.0 drinks/d | ≥3.5 drinks/d |  |  |  |
| EPA + DHA |  |  |  | 10 | 250 mg/d | 0 |  |  |  |
| PUFA |  |  |  | 10 | **≥**10% of energy | ≤2% of energy |  |  |  |

AHEI, alternate Healthy Eating Index; CHEI, Chinese Healthy Eating Index; DASH, Dietary Approach to Stop Hypertension; DHA, Docosahexaenoic acid; EPA, Eicosapentaenoic acid; PUFA, polyunsaturated fatty acid; Max, maximum; Min, minimum; Q5, highest quintile; Q1, lowest quintile; SP/1000 kcal, Standard Portion per 1000 kcal;

^a.^ CHEI score has been reproduced from Yuan Y-Q., et al., The Development of a Chinese Healthy Eating Index and Its Application in the General Population. Nutrients, 2017. 9(9).

^b.^ AHEI score has been reproduced from Chiuve SE, et al. Alternative dietary indices both strongly predict risk of chronic disease. The Journal of nutrition, 2012, 142(6)

^c.^ DASH score has been reproduced from Fung TT., et al., Adherence to a DASH-style diet and risk of coronary heart disease and stroke in women. Archives of internal medicine, 2008, 168(7)

^d.^ Sugar-sweetened beverages, include carbonated drinks and juice;

^e.^ Since the average daily salt intake of participants was not asked in the FFQ, we modified the scoring criteria of salt intake. We scored the daily salt intake according to the item of FFQ, “What are the saltiness and lightness of your dishes?”. “Very salty” is scored 5 points, “salty” is scored 4 points, “almost” is scored 3 points, “light” is scored 2 points, and “very light” is scored 1 point.

^f.^ Since the average daily oil intake of participants was not asked in the FFQ, we modified the scoring criteria of oil intake. We scored the daily oil intake according to the item of FFQ, “What is the amount of oil for your dishes?”. “Very much” is scored 5 points, “relatively much” is scored 4 points, “almost” is scored 3 points, “relatively little” is scored 2 points, and “very little” is scored 1 point.

**Supplementary Table 2.** The characteristics of participants by tertiles of CHEI score

| **Characteristics** | **Tertiles of scores** | | | ***P**** |
| --- | --- | --- | --- | --- |
|  | **T1** | **T2** | **T2** |  |
| **Age (years)** | 33.58 ± 5.36 | 32.26 ± 4.70 | 32.16 ± 4.65 | < 0.01 |
| **Body mass index (kg/m^2^)** | 26.22 ± 4.60 | 26.10 ± 3.95 | 26.06 ± 4.53 | 0.65 |
| **Physical activity (MET/hours/week)** | 160.65 ± 97.86 | 168.25 ± 103.26 | 168.09 ± 111.68 | 0.39 |
| **Abstinence time (days)** | 4.31 ± 1.39 | 4.52 ± 1.45 | 4.22 ± 1.40 | 0.45 |
| **Energy intake (kcal/day)** | 1918.54 ± 636.32 | 1767.50 ± 524.23 | 1651.38 ± 499.73 | < 0.01 |
| **Educational level (*n,* %)** |  |  |  | < 0.01 |
| Middle school or below | 94 (31.76) | 100 (33.90) | 132 (44.59) |  |
| College or higher | 202 (68.24) | 195 (66.10) | 164 (55.41) |  |
| **Annual income (RMB,** thousand yuan**) (*n,* %)** | |  |  | < 0.05 |
| <50 | 31 (10.47) | 41 (13.90) | 61 (20.61) |  |
| 50 to <100 | 115 (38.85) | 117 (39.66) | 110 (37.16) |  |
| ≥100 | 150 (50.68) | 137 (46.44) | 125 (42.23) |  |
| **Current smoker (*n,* %)** | 115 (38.85) | 140 (47.46) | 188 (63.51) | < 0.01 |
| **Current drinker (*n,* %)** | 89 (30.07) | 128 (43.39) | 148 (50.00) | < 0.01 |
| **Semen parameters** |  |  |  |  |
| Ejaculate volume (ml) | 3.55 ± 1.42 | 3.62 ± 1.37 | 3.42 ± 1.36 | 0.23 |
| Sperm concentration (10^6^/ml) | 63.89 ± 41.56 | 64.95 ± 42.01 | 62.83 ± 37.05 | 0.75 |
| Total sperm count (10^6^/ml) | 216.15 ± 152.25 | 222.57 ± 153.63 | 203.87 ± 124.73 | 0.30 |
| Progress motility (%) | 35.33 ± 15.41 | 35.86 ± 15.37 | 39.27 ± 14.20 | < 0.01 |
| Total motility (%) | 44.07 ± 18.94 | 44.25 ± 18.57 | 48.42 ± 17.15 | < 0.01 |
| Normal sperm morphology (%) | 5.06 ± 3.25 | 5.04 ± 3.35 | 5.51 ± 3.16 | 0.10 |

CHEI, Chinese Healthy Eating Index; MET, metabolic equivalent.

Values are presented as mean ± SD (continuous variables) or as *n* and % (categorical variables).

* P value was determined by chi-square test.

**Supplementary Table 3.** The characteristics of participants by tertiles of AHEI-2010 score

| **Characteristics** | **Tertiles of scores** | | | ***P**** |
| --- | --- | --- | --- | --- |
|  | **T1** | **T2** | **T2** |  |
| **Age (years)** | 33.52 ± 5.45 | 32.84 ± 4.78 | 31.63 ± 4.40 | < 0.01 |
| **Body mass index (kg/m^2^)** | 26.40 ± 4.19 | 25.99 ± 4.19 | 25.98 ± 4.69 | 0.24 |
| **Physical activity (MET/hours/week)** | 161.86 ± 104.89 | 165.56 ± 99.00 | 169.56 ± 109.15 | 0.37 |
| **Abstinence time (days)** | 4.40 ± 1.39 | 4.34 ± 1.45 | 4.31 ± 1.42 | 0.47 |
| **Energy intake (kcal/day)** | 2047.40 ± 663.39 | 1643.05 ± 470.02 | 1646.55 ± 442.60 | < 0.01 |
| **Educational level (*n*, %)** |  |  |  | 0.16 |
| Middle school or below | 99 (33.45) | 106 (35.93) | 121 (40.88) |  |
| College or higher | 197 (66.55) | 189 (64.07) | 175 (59.12) |  |
| **Annual income (RMB,** thousand yuan**) (*n*, %)** |  |  |  | 0.57 |
| <50 | 42 (14.19) | 42 (14.24) | 49 (14.19) |  |
| 50 to <100 | 117 (39.53) | 106 (35.93) | 119 (40.20) |  |
| ≥100 | 137 (46.28) | 147 (49.83) | 128 (43.24) |  |
| **Current smoker (*n*, %)** | 118 (60.14) | 145 (50.85) | 180 (39.19) | < 0.01 |
| **Current drinkers (*n*, %)** | 112 (62.16) | 104 (64.75) | 149 (49.66) | < 0.01 |
| **Semen parameters** |  |  |  |  |
| Ejaculate volume (ml) | 3.54 ± 1.48 | 3.54 ± 1.33 | 3.52 ± 1.34 | 0.83 |
| Sperm concentration (10^6^/ml) | 63.80 ± 42.28 | 65.55 ± 41.10 | 62.34 ± 37.22 | 0.65 |
| Total sperm count (10^6^/ml) | 214.41 ± 152.62 | 219.9 ± 141.76 | 208.27 ± 138.02 | 0.61 |
| Progress motility (%) | 34.43 ± 15.59 | 36.94 ± 14.99 | 39.09 ± 14.35 | < 0.01 |
| Total motility (%) | 42.87 ± 19.06 | 45.86 ± 18.19 | 48.01 ± 17.39 | < 0.01 |
| Normal sperm morphology (%) | 4.94 ± 3.28 | 5.23 ± 3.20 | 5.44 ± 3.27 | 0.07 |

AHEI, alternate Healthy Eating Index; MET, metabolic equivalent.

Values are presented as mean ± SD (continuous variables) or as *n* and % (categorical variables).

* P value was determined by chi-square test.

**Supplementary Table 4.** The characteristics of participants by tertiles of DASH score

| **Characteristics** | **Tertiles of scores** | | | ***P**** |
| --- | --- | --- | --- | --- |
|  | **T1** | **T2** | **T2** |  |
| **Age (years)** | 33.18 ± 5.32 | 32.62 ± 4.5 | 32.13 ± 4.76 | < 0.01 |
| **Body mass index (kg/m^2^)** | 26.19 ± 4.51 | 26.17 ± 4.09 | 26.03 ± 4.39 | 0.63 |
| **Physical activity (MET/hours/week)** | 161.85 ± 97.64 | 154.84 ± 98.12 | 176.72 ± 114.18 | 0.06 |
| **Abstinence time (days)** | 4.36 ± 1.41 | 4.29 ± 1.46 | 4.38 ± 1.41 | 0.81 |
| **Energy intake (kcal/day)** | 2029.43 ± 644.19 | 1734.13 ± 492.16 | 1535.37 ± 376.77 | < 0.01 |
| **Educational level (*n*, %)** |  |  |  | < 0.01 |
| Middle school or below | 116 (32.77) | 61 (29.33) | 149 (45.85) |  |
| College or higher | 238 (67.23) | 147 (70.67) | 176 (54.15) |  |
| **Annual income (RMB,** thousand yuan**) (*n*, %)** |  |  |  | 0.41 |
| <50 | 43 (12.15) | 35 (16.83) | 55 (16.92) |  |
| 50 to <100 | 141 (39.83) | 81 (38.46) | 120 (36.92) |  |
| ≥100 | 170 (48.02) | 92 (44.23) | 150 (46.15) |  |
| **Current smoker (*n*, %)** | 142 (40.11) | 95 (45.67) | 206 (63.38) | < 0.01 |
| **Current drinker (*n*, %)** | 129 (36.44) | 80 (38.46) | 156 (48.00) | < 0.01 |
| **Semen parameters** |  |  |  |  |
| Ejaculate volume (ml) | 3.55 ± 1.41 | 3.57 ± 1.48 | 3.49 ± 1.29 | 0.58 |
| Sperm concentration (10^6^/ml) | 64.59 ± 42.79 | 62.96 ± 38.26 | 63.72 ± 38.66 | 0.78 |
| Total sperm count (10^6^/ml) | 215.80 ± 149.59 | 215.29 ± 142.9 | 211.73 ± 139.34 | 0.71 |
| Progress motility (%) | 35.39 ± 15.42 | 36.05 ± 14.48 | 38.87 ± 14.92 | < 0.01 |
| Total motility (%) | 43.90 ± 18.77 | 44.77 ± 17.77 | 47.93 ± 17.99 | < 0.01 |
| Normal sperm morphology (%) | 4.99 ± 3.22 | 5.31 ± 3.4 | 5.38 ± 3.20 | 0.13 |

DASH, Dietary Approach to Stop Hypertension; MET, metabolic equivalent.

Values are presented as mean ± SD (continuous variables) or as *n* and % (categorical variables).

* P value was determined by chi-square test
